# Evaluation of the Wound Healing Potential of Some Natural Polymers on Three Experimental Models

**DOI:** 10.3390/ph14050465

**Published:** 2021-05-14

**Authors:** Calin Vasile Andritoiu, Corina Elena Andriescu, Maricel Danu, Cristina Lungu, Bianca Ivanescu, Cornel Havarneanu, Marcel Popa

**Affiliations:** 1 Apitherapy Medical Center, Balaneşti, 217036 Gorj, Romania; dr_calin_andritoiu@yahoo.com; 2 Nutrition and Dietetics Specialization, Faculty of Pharmacy, Vasile Goldis Western University of Arad, 310025 Arad, Romania; 3 Department of Pathology, Sf. Spiridon Emergency County Hospital, 700111 Iasi, Romania; andriescu_corina@yahoo.co.uk; 4 Petru Poni Institute of Macromolecular Chemistry, 700487 Iasi, Romania; mdanu@tuiasi.ro; 5 Department of Natural and Synthetic Polymers, Cristofor Simionescu Faculty of Chemical Engineering and Environmental Protection, Gheorghe Asachi Technical University of Iasi, 700050 Iasi, Romania; marpopa2001@yahoo.fr; 6 Department of Pharmaceutical Botany, Faculty of Pharmacy, Grigore T. Popa University of Medicine and Pharmacy, 700115 Iasi, Romania; 7 Faculty of Psychology and Education Sciences, Alexandru Ioan Cuza University, 700554 Iasi, Romania; hcornel@uaic.ro; 8 Academy of Romanian Scientists, 050094 Bucharest, Romania

**Keywords:** natural polymers, chitosan, collagen, ovalbumin, egg white, wound healing, rheological characterization

## Abstract

The aim of this paper was the preparation and investigation of the wound healing properties of four topical formulations based on natural polymers such as collagen, chitosan, lyophilized egg white, and a mixture of them. The therapeutic assessment of these four ointments was carried out in vivo on the incision, excision, and thermal burn wounds induced on Wistar rats. The treatment was applied topically on wounds once a day, for 21 days. The experimental results were analyzed from a clinical and histopathological point of view. The rheological characterization of the topical formulations was also performed in order to verify their spreadability and structural stability. All ointments had a positive effect on wound contraction and re-epithelization processes, but the one based on total polymers had a significant healing potential on the designed cutaneous lesions due to its synergistic effects.

## 1. Introduction

The human body survives in the environment due to a dynamic balance called the homeostasis process. The skin has a major role in maintaining homeostasis, being a protective barrier between the body and the environment [1]. Every day, the skin is exposed to multiple physical and thermal aggressions and the cutaneous lesions need a specialized medical service that usually implies a high cost [2]. Finding less traumatic methods of treatment is imperiously required and the final goal in wound healing research is to achieve a cure through a regeneration process. The dermis contains a series of proteins of which the collagen polymer, responsible for the tensile strength of the skin, has an important percentage (~70–80% of the dry weight) [3].

Collagen is a fibrous material entity that has a specific three-dimensional conformation–triple helix structure–with high mechanical strength and good cell recognition [4,5]. Collagen-based materials have numerous applications in the tissue regeneration field due to their biocompatibility properties. On the other hand, this polymer is one of the main materials present in the architecture of the living matter and can be efficiently extracted from animal’s tissues such as skin, bones, and tendons [3].

Chitosan belongs to a class of polymers widespread in nature, based on glucosamine monomers, known for their antimicrobial activity. Chitosan is a non-toxic hydrophilic polymer obtained by N-deacetylation of chitin in the presence of 40–50% NaOH at 180 °C [5]. It is widely used in pharmaceutical, cosmetic, and food industries due its antioxidant, antifungal, antibacterial and hemostatic properties [6]. In the human body, chitosan is degraded by an enzyme called lysozyme, leading to degradation products that are able to stimulate macrophages, positively influence collagen sedimentation and, consequently, accelerate the healing of wounds [4,7]. 

Another natural polymer is the ovalbumin found in lyophilized egg white. Due to its property to create a protective film on the surface of the wound, it is a good choice for treating skin lesions [8].

In this context, our paper aimes to formulate four ointments based on different polymers (CH–chitosan, CO–collagen, AL–ovalbumin from lyophilized egg white of *Gallus gallus domesticus*, and POL–a mixture of CH, CO and AL) and to investigate their wound healing potential in vivo on incision, excision, and thermal burn wounds models. The inclusion of polymers in the ointments formulations has a very important effect on the spreadability and, of course, on the structural properties of the pharmaceutical form. In order to provide a good description of these aspects, rheological analyses were performed. Thus, the ointment formulations were studied using the oscillatory tests (amplitude sweep, frequency sweep, and temperature test) and rotational tests (flow curves).

## 2. Results

### 2.1. Rheological Characterization

**Figure 1:**
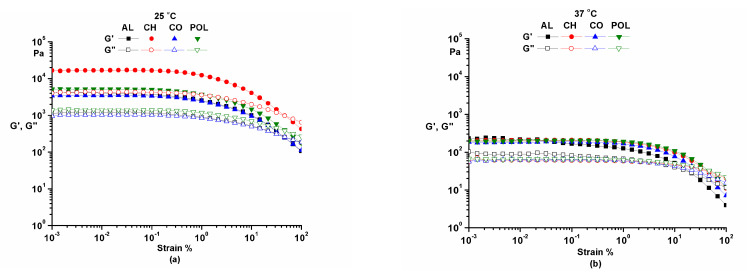
Amplitude sweep at 25 °C (**a**) and 37 °C (**b**).

To determine the linear viscoelastic range and the structure stability of samples, the amplitude sweep test (γ= 0.001–100%, ω = 10 rad/s) was performed (Figure 1). 

**Figure 2:**
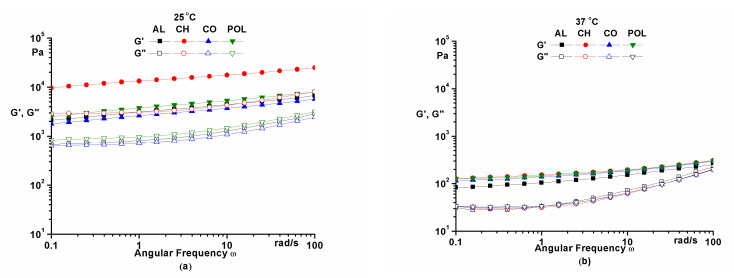
Frequency sweep at 25 °C (**a**) and 37 °C (**b**).

The frequency sweep (Figure 2) was performed at a constant amplitude (in the limit of the linear viscoelastic range γ = 0.01% at 25 °C and γ = 0.05% at 37 °C). The angular frequency varied in the domain 0.1 ÷ 100 rad/s.

**Figure 3:**
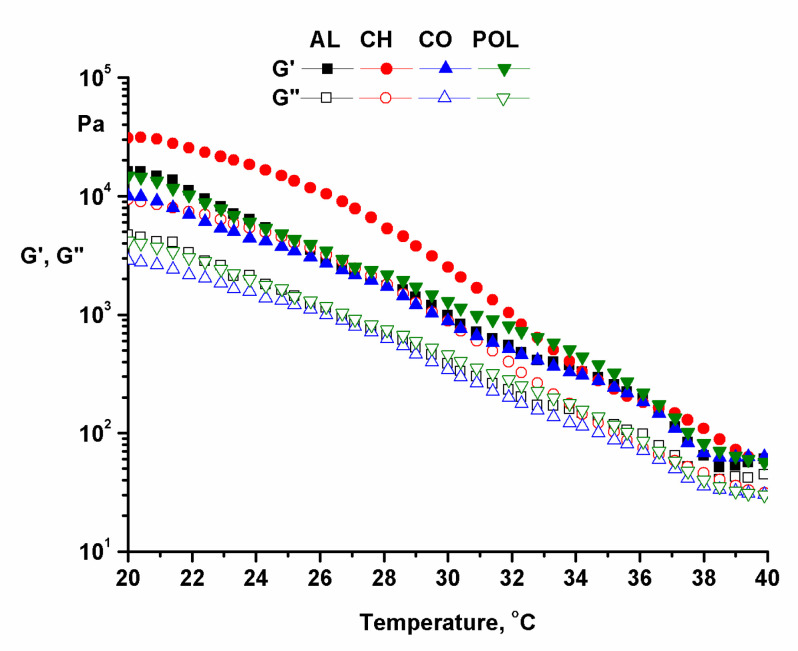
Temperature test in oscillation mode.

Temperature test was performed at a constant frequency (ω = 10 rad/s) and a constant amplitude in the linear viscoelastic range (γ = 0.01%) with a temperature variation in the range 20–40 °C (Figure 3).

**Figure 4:**
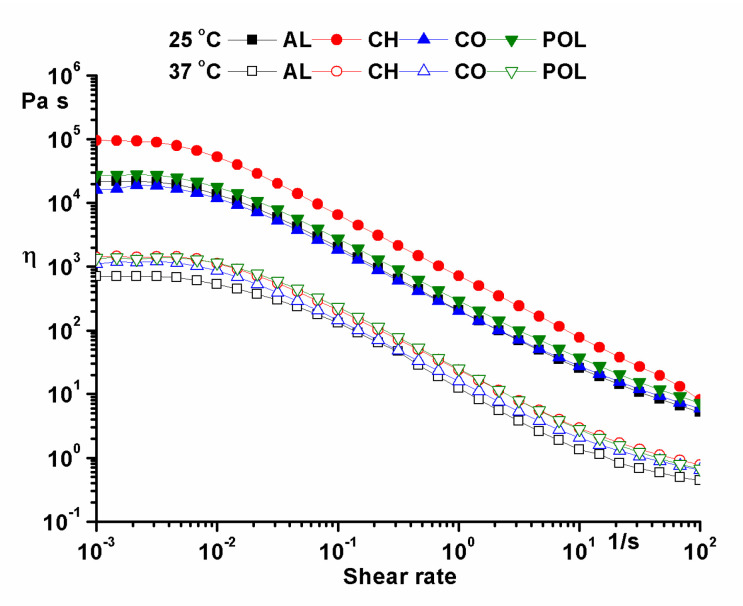
Temperature test in oscillation mode.

Rotational tests (Figure 4–flow curves) were performed using a shear variation between 0.001 and 100 s^−1^. The values of zero shear viscosity are listed in Table 1.

### 2.2. Wound Healing Evaluation Parameters

#### 2.2.1. Period of Re-epithelialization and Wound Contraction Rate

The wound areas were measured at days 0, 6 and 9. The results are presented in Table 2. Because in the first three days of treatment the inflammatory processes are present, there were no important changes in terms of the wound area. The cell proliferation process started on the 3rd day and a significant reduction in wound area (*p* < 0.001) was obtained on the 6th and 9th days. The best results were recorded for the group treated with lyophilized egg white (1 × 2 mm^2^) on day 9, followed by the group treated with chitosan (2 × 2 mm^2^). 

The period of re-epithelialization was determined in relation with the days required for the falling of scabs. This time was taken at the end-point of complete epithelialization which was observed on day 12 for all groups treated with natural polymers in the case of the excision-type lesion (Table 2). The re-epithelialization process finished on day 18 for OB group and on day 21 for NC group. 

In terms of the wound contraction rate, on the 6th day of the experiment, it was observed, that treatment with lyophilized egg white ointment had the best effects on the wound contraction rate (WCR = 64.06 ± 0.45%), as compared to the group treated with ointment base (WCR = 26.74 ± 2.13%), and, in particular, with the NC group (WCR = 5.78 ± 1.86%) (Table 2). Moreover, on the 9th day of the treatment, the results were obviously positive for AL group (WCR = 97.14 ± 0.14%), followed by CH group (WCR = 93.43 ± 0.33%) and POL group (WCR = 91.46 ± 0.45%).

Macroscopic pictures regarding wound contraction rate, as well as for the incision-type lesion and thermal burn are presented in Figure S1.

#### 2.2.2. Histological Examination 

**Figure 5:**
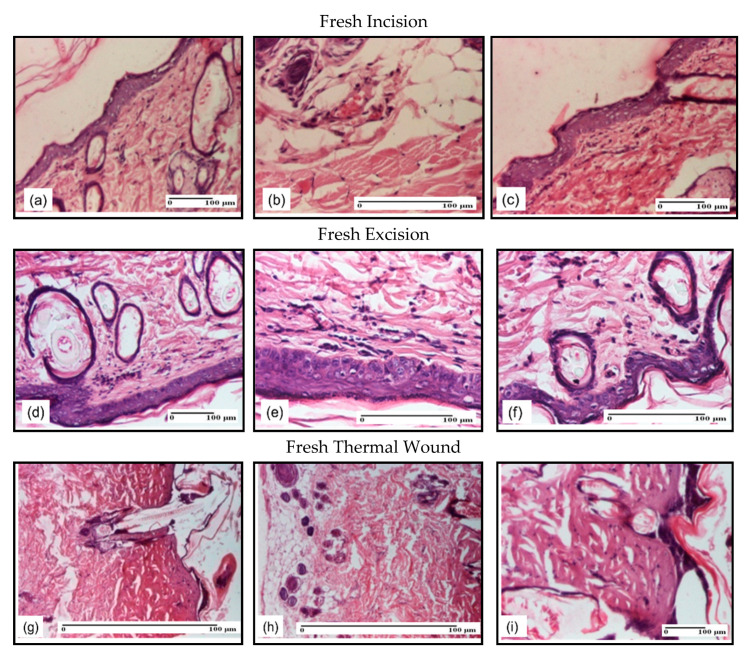
Microscopic histology images of fresh cutaneous wounds models: (**a**) Rare lymphocytes (S1); (**b**) Vessel congestion (S1); (**c**) Hemorrhage in the superficial dermis (S1); (**d**) Epidermis and dermis with dermal edema and hair follicle ectasia (S2); (**e**) Light inflammatory infiltrate in the superficial dermis and slight dermal edema (S2); (**f**) Slight dermal edema and dilatation of the hair follicles (S1); (**g**) Severe dermal collagenization (S1, D1); (**h**) Severe dermal collagenization (S1, D1); (**i**) Severe dermal collagenization (S1, D1). Inflammatory infiltration scoring: S0 (no inflammatory infiltrate), S1 (mild inflammatory infiltrate), S2 (moderate inflammatory infiltrate), S3 (severe inflammatory infiltrate). The thermal burn depths (D): D0—normal skin, D1—epithelial necrosis within the epidermis, the basement membrane remains intact, D2—necrosis of skin appendages and dermal connective tissue; D3—extensive necrosis within the hypodermic tissue.

In the sampling day, all cutaneous lesions presented: 1–2 lymphocytes/high-power field (HPF) and 3–4 fibroblasts/HPF with one capillary vessel/HPF and local micro hemorrhage, mild edema, moderate collagen denaturation in incision and excision wounds, localized at the superficial dermis, and severe collagen denaturation in the thermal burns, as shown in Figure 5.

**Figure 6:**
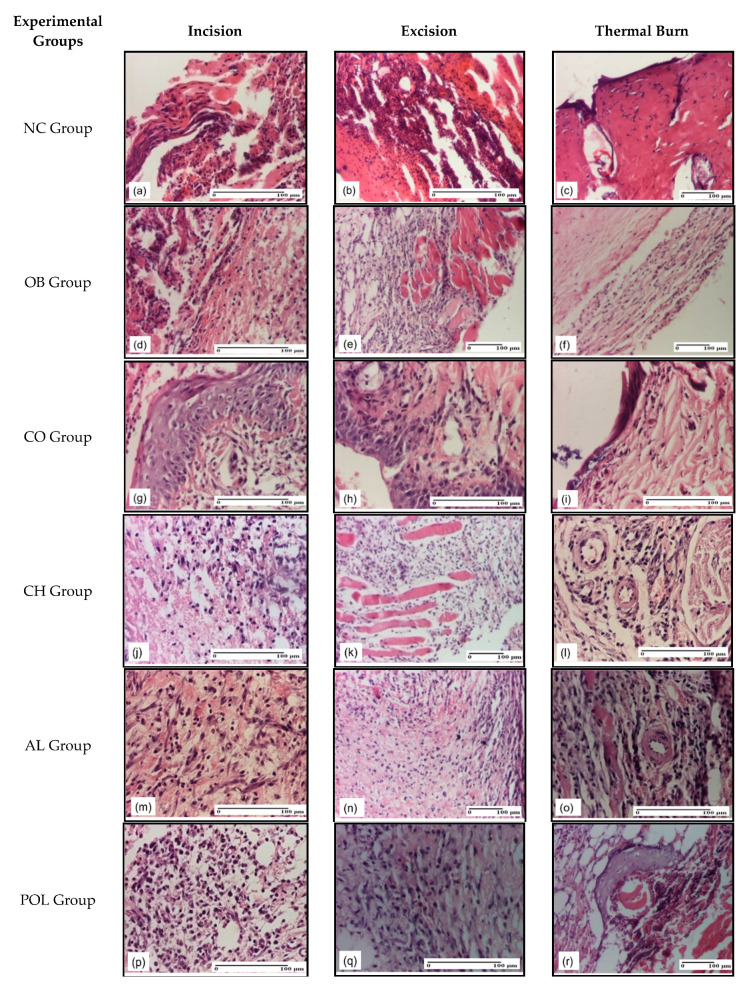
Microscopic histology images on day 3: (**a**) Abscess in keratin layer (S3); (**b**) Ulcerated epidermis with inflammation in dermis and hypodermis (lymphocytic deposit) (S3); (**c**) Significant dermal collagenization (S1, D1); (**d**) Congestion and inflammatory infiltrate in dermis (S3); (**e**) Inflammation in hypodermis, muscular layer (S3); (**f**) Inflammation (S2, D3); (**g**) Inflammatory infiltrate (S2); (**h**) Areas of regenerated epidermis, inflammation in hypodermis (S2); (**i**) Perivascular lymphocytes (S1, D1); (**j**) Inflammation in dermis (S2); (**k**) Important inflammation (S3); (**l**) Inflammation in hypodermis (S2, D1); (**m**) Granulation tissue undergoing connective organization, lymphocytes (S1); (**n**) Granulation tissue with inflammatory infiltrate in hypodermis (S2); (**o**) Inflammatory infiltrate (S2, D1); (**p**) Important inflammatory infiltrate in hypodermis (S3); (**q**) Granulation tissue (S2); (**r**) Perivascular inflammatory infiltrate (S2, D2); Inflammatory infiltration scoring: S0 (no inflammatory infiltrate), S1 (mild inflammatory infiltrate), S2 (moderate inflammatory infiltrate), S3 (severe inflammatory infiltrate); The thermal burn depths (D): D0–normal skin, D1–epithelial necrosis within the epidermis, the basement membrane remains intact, D2–necrosis of skin appendages and dermal connective tissue; D3—extensive necrosis within the hypodermic tissue.

On the 3rd day of treatment (Figure 6), similar aspects were found in all three models, with intracorneal abscess and ulcerative area of the epidermis, more pronounced for the untreated group (NC) (Figure 6a–c). For this group, in the case of incision, a massive abscess in the keratin layer was observed, as well as ulceration of the epidermis with inflammation in the dermis and the hypodermis and massive lymphocytic deposit. Aspects of dermal collagenization were highlighted in the thermal burn lesion (Figure 6a–c).

For the group treated with ointment base (OB Group), congestion and important inflammatory infiltrate were found in the dermis in the incision model, while for the excision model, inflammation of the hypodermis that descends to the muscular plane was noted. In case of thermal burning lesion, inflammation was present (Figure 6d–f). 

The inflammation of the hypodermis correlated with hemorrhage (incision and thermal burns models) and fibrosis around the muscular fascicles (excision and thermal burns models) were the findings for the CO group (Figure 6g–i).

The group treated with chitosan (CH group) revealed inflammation of the epidermis and dermis (incision lesion), inflammation of the hypodermis (thermal burn lesion), and inflammation of the muscular layer (excision lesion) (Figure 6j–l).

As for the group treated with lyophilized egg white (AL group), we have noted the following aspects in the incision, excision, and thermal burn models: mixed inflammatory infiltrate at the level of the hypodermis, moderate in incision and excision and severe in thermal burn. We also observed the inflammation of the muscle fragment, moderate in excision and severe in the thermal burning, and mature granulation tissue. In the excision model, the granulation tissue was maturing, which also affects the submuscular tissue, leading to bleeding and associated necrosis. 1–2 lymphocytes, respectively fibroblasts/HPF with moderate edema and epidermis without changes have been present at the superficial dermis. In addition, severe fibrosis of the superficial dermis was observed in the thermal burn (Figure 6m–o).

On the 3rd day, the remarks concerning the group treated with ointment based on natural polymers (POL group) were classified as follows: for the incision-like lesion, we have noted unaffected epidermis and perivascular inflammatory infiltrate in the dermis, together with the appearance of maturing granulation tissue in the hypodermis and muscle layer. At the level of burn-type injury we have observed an important inflammatory infiltrate into the hypodermis (Figure 6p–r).

**Figure 7:**
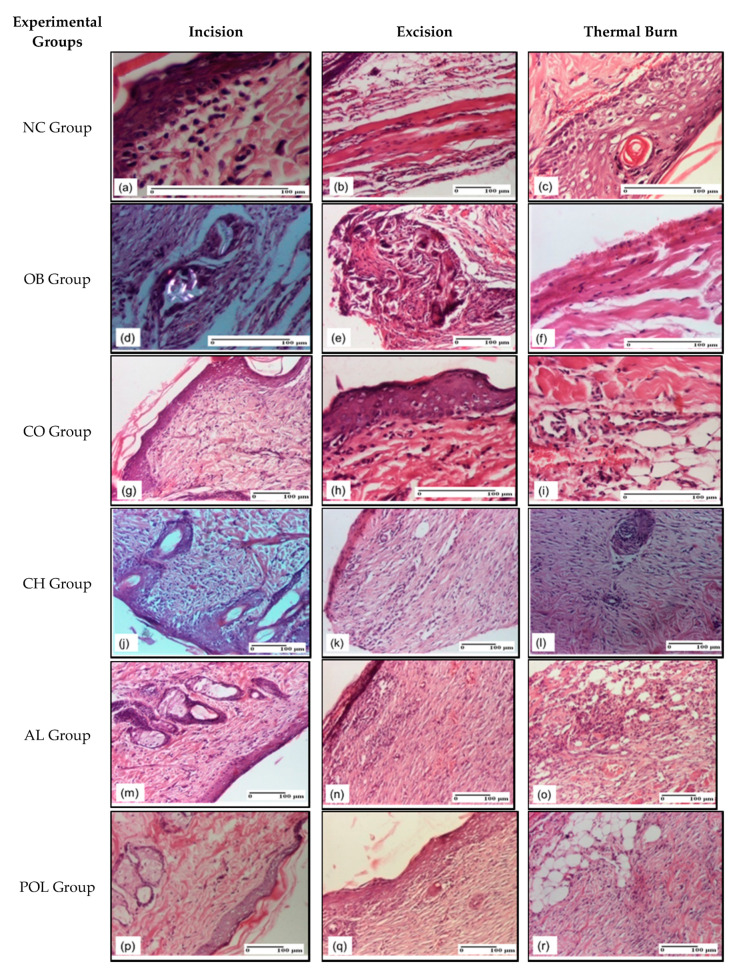
Microscopic histology images on day 9: (**a**) Inflammatory infiltrate in superficial dermis (S1); (**b**) Inflammatory infiltrate in muscular layer (S2); (**c**) Vacuolar degeneration (S1, D0); (**d**) Multinucleated foreign-body giant cells, detail in polarized light microscopy (S2); (**e**) Multinucleated foreign-body giant cells (S2); (**f**) Rare inflammatory infiltrate in muscular layer (S1, D0); (**g**) Dermal collagenization, inflammatory infiltrate in superficial dermis (S1); (**h**) Moderate inflammatory infiltrate in superficial dermis (S1); (**i**) Inflammation in hypodermis (S1, D0); (**j**) Slight dermal collagenization (S2); (**k**) Dermal collagenization (S2); (**l**) Significant dermal collagenization as result of granulation tissue maturation (S1, D0); (**m**) Mature granulation tissue (S1); (**n**) Dermal collagenization (S2); (**o**) Granulation tissue undergoing connective organization associated with important lymphoplasmacytic inflammatory infiltrate (S2, D2); (**p**) Significant dermal collagenization, rare lymphocytes (S1); (**q**) Significant dermal collagenization due to the maturation of granulation tissue (S1); (**r**) Significant dermal collagenization (S2, D0). Inflammatory infiltration scoring: S0 (no inflammatory infiltrate), S1 (mild inflammatory infiltrate), S2 (moderate inflammatory infiltrate), S3 (severe inflammatory infiltrate). The thermal burn depths (D): D0–normal skin, D1–epithelial necrosis within the epidermis, the basement membrane remains intact, D2–necrosis of skin appendages and dermal connective tissue; D3–extensive necrosis within the hypodermic tissue.

On the 9th day of the experiment (Figure 7), in the group treated with collagen ointment a similar behavior was observed for the three model types in terms of the number of lymphocytes, 2–3/HPF and fibroblasts, 2–3/HPF, in both incision and excision, and 4–5/HPF in the thermal burn; other observations are the similar number of capillaries in the superficial dermis, 1–2/HPF for incision and excision and 3/HPF in the thermal burn; the next notes concern the moderate edema in incision and excision and mild edema in the thermal burn. Discrete dermal collagenization was noticed in incision and excision and severe colagenization in thermal burn, with the homogenization of collagen bundles, especially in the deep dermis. In regards to the number of cell layers (3–4) with rectilinear areas, the epidermis was within normal limits (Figure 7g–i).

For the group treated with chitosan, on the 9th day, our results showed that there were 2–3 lymphocytes/HPF, respectively fibroblasts, in incision and excision, and 3–4 lymphocytes, respectively fibroblasts/HPF, in the thermal burn. The epidermis did not present any microscopic changes. Also, within incision and excision, mature granulation tissue and dermal collagenization were observed (Figure 7j–l).

For the three experimental models treated with lyophilized egg white, mature granulation tissue was revealed from the epidermis to the hypodermis, where inflammatory elements (lymphocytes) were also present (Figure 7m–o).

On the 9th day, for the POL group, the observations show significant dermal collagenization for both incision and excision, due to the maturation of granulation tissue (mature granulation tissue to the hypodermis). Rare lymphocytes were found. The results in excision and incision were: 2–3 lymphocytes/HPF and 2–3 fibroblasts/HPF; 2–3 capillaries/HPF in excision and one capillary vessel/HPF in incision model. There were no epidermal changes. As for thermal burn, there was a significant dermal collagenization (Figure 7p–r).

For the group treated with ointment base, the presence of giant foreign body cells was noted in keratin. This feature is common to all three experimental models. For the NC group, the presence of inflammatory infiltrate was noticed in the superficial dermis in the incision type lesion. As for the excision type lesion, it was seen that the inflammatory infiltrate also affected the deep planes, including the muscular layer. Also, vacuolar degeneration was found in the thermal burn (Figure 7d–f).

**Figure 8:**
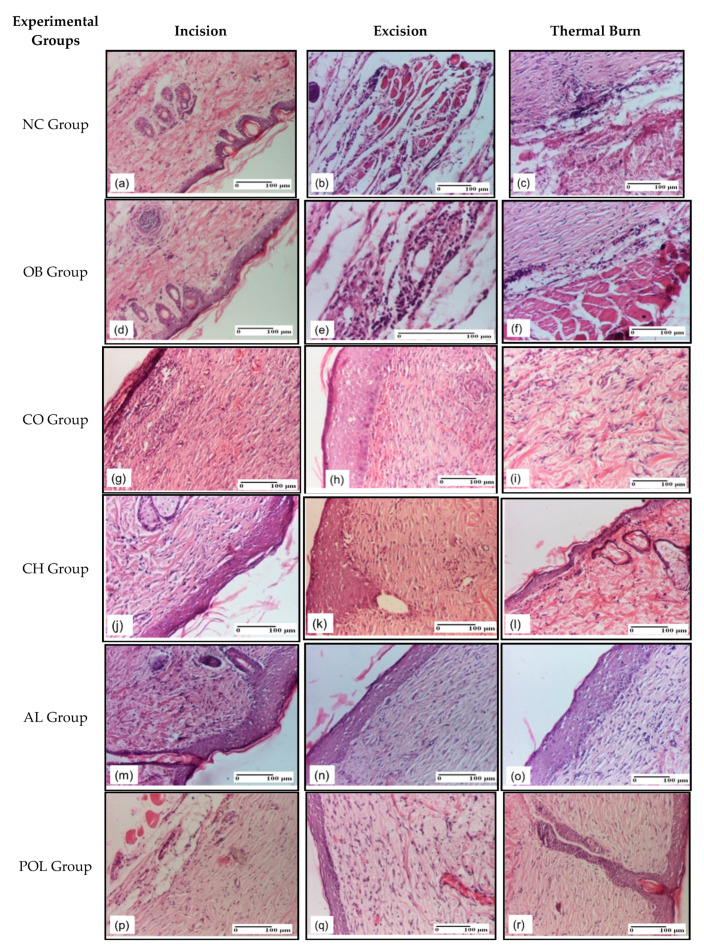
Microscopic histology images on day 21: (**a**) Edema (S1); (**b**) Inflammation in striated muscle (S2); (**c**) Inflammatory infiltrate (S2, D0); (**d**) Edema (S1); (**e**) Inflammatory infiltrate in hypodermis (S2); (**f**) Inflammatory infiltrate (S2, D0); (**g**) Mature granulation tissue (S2); (**h**) Significant dermal collagenization (S1); (**i**) Dermal collagenization (S1, D0); (**j**) Dermal collagenization (S1); (**k**) Dermal collagenization (S1); (**l**) Dermal collagenization (S1, D0); (**m**) Dermal collagenization (S1); (**n**) Mature granulation tissue, dermal collagenization (S1); (**o**) Significant dermal collagenization due to the maturation of granulation tissue (S2, D0); (**p**) Significant dermal collagenization (S1); (**q**) Slight dermal collagenization (S1); (**r**) Significant dermal collagenization (S1, D0). Inflammatory infiltration scoring: S0 (no inflammatory infiltrate), S1 (mild inflammatory infiltrate), S2 (moderate inflammatory infiltrate), S3 (severe inflammatory infiltrate). The thermal burn depths (D): D0–normal skin, D1–epithelial necrosis within the epidermis, the basement membrane remains intact, D2–necrosis of skin appendages and dermal connective tissue; D3–extensive necrosis within the hypodermic tissue.

On the 21st day of the experiment (Figure 8), a significant dermal collagenization for both the incision type lesion and the thermal burn lesion was observed for the group treated with ointment based on natural polymers, and a slight dermal collagenization in the excision type lesion (Figure 8p*–*r). The groups treated with polymers tested separately also showed positive results, as they presented important dermal collagenization at that time (Figure 8g*–*o).

On the other hand, for the group treated with ointment base, the presence of congestion was noticed in the incision type lesion and dermal edema in the excision type lesion. Also, the examination of the samples indicated that lymphocytes are present in the thermal burn (Figure 8d*–*f). Moreover, congestion was observed for the untreated group in the incision-type lesion, along with dermal edema and perivascular inflammatory infiltrate in the excision-type lesion. The inflammatory infiltrate was also present in the thermal burn lesion (Figure 8a*–*c).

## 3. Discussion

The spreadability and structural stability are important parameters of the ointments. The inclusion of polymers in the ointment formulation has a significant influence on these properties. Useful information about spreadability and structural stability can be obtained by performing oscillatory and rotational rheological tests. The reproducibility of the results was verified by performing rheological tests on three samples of each ointment.

The amplitude sweep was used to determine the limit of the linear viscoelastic range (γ = 0.01–0.05%). This test confirmed the structural stability of the samples at small deformations for both temperatures (25 °C and 37 °C). The samples presented a high rigidity and the structure was more sensitive to deformations at 25 °C. At a physiological temperature (37 °C), the sample structure was flexible due to the slight decrease of the viscosity and the increase of polymer chain flexibility. Therefore, at a physiological temperature, the ointments offered a good sensation and a good spreadability on the skin. 

The frequency sweep test highlighted the solid-like behavior of the sample at both temperatures due to the higher G’ (storage modulus) values than G” (loss modulus) over the entire domain of frequency. The parallel dynamic modules and their small frequency variation suggested the presence of a stable tridimensional network. At a physiological temperature, the values of the dynamic modules are lower than at 25 °C suggesting a smooth feeling and a good spreadability on the skin.

The structural stability and the degree of fluidization of the ointments at physiological temperature were both highlighted using the temperature test. Obviously, the values of the dynamic moduli decreased with increasing temperature, as explained above.

The high values of zero shear viscosity calculated with the Carreau-Yasuda model using the flow curves also emphasized the structural stability of the samples [9,10,11].

Considering that burn injuries are a major cause of morbidity and mortality worldwide [12], the accurate description of the microscopic pathological aspects of these lesions aims to understand the healing processes [12,13,14] and consequently therapeutic efficacy. As mentioned by some researchers [12,13,14], there is still a need to develop studies for the integration of the healing process in the current therapeutic context. Thus, using different materials, some research teams [12,13,14] managed to rank burns/injuries based on the design of scoring scales that show the extent of these lesions.

Using the same scoring systems, but under different study conditions, our results enabled the comparative analysis of different study models. Thus, the scoring of the inflammatory process allowed for the highlight of the reduction of inflammation in all models analyzed in relation to the day on which the biopsy was performed (day 3, 9 and 21, respectively) with downward scores from S3/S2 on day 3 to S1 on day 9 and day 21, respectively. This reaction pattern is characteristic of the NC, OB, CH and POL groups respectively for the incision model. For the excision category, the scores are decreasing, often by one degree (from S3 to S2 for the NC, OB groups, or from S2 to S1 for the CO, AL, and POL groups). The values of the scores are therefore relatively close for the groups used (NC, OB, CO, CH, AL, POL) and there is a favorable evolution for each category. Also, the depth of the burn (D) shows a favorable evolution, from D1 to D0 in the groups NC, CO, CH, POL, the only group evolving from D1 to D2 being AL on day 9 and with regression to D0 on day 21, thus demonstrating a favorable finality.

The inclusion of natural polymers in the tested ointments was based on the idea that the response of biological media (i.e., living tissues: traumatic wounds or various types of surgical wounds) to their contact with collagenous materials is embodied by specific phenomena: hemostasis, immunological response and cell proliferation, as presented in scientific literature [15]. The hemostatic activity, one of the most important biological properties of collagen, causes the blood to coagulate when it comes in contact with it: platelets adhere in the first phase to the surface of the collagen gel, then they desorb and become involved in the aggregation process and next, they activate the thrombin reaction on fibrinogen, which ends with the appearance of platelet thrombus [16]. Collagenous materials also have the property of generating new tissue [17], which was demonstrated in the present study.

Collagen is involved in wound healing due to its integrin binding domains, which promote cell attachment, a phenomenon necessary for cell growth, differentiation and metabolic activity [4,18]. 

Collagen is naturally degraded by metalloproteinases, specifically collagenases and serine proteases, so that its degradation is under the local control of cells in cultured tissue [3]. Literature data also mention collagen and chitosan-derived hydrogels used in the treatment of wounds due to hemostatic and antimicrobial effects, correlated with good biocompatibility [19].

In fact, the chitosan ointment used in the present experiment proved to have an important role in the regeneration of the affected tissue, which was confirmed by previous studies [6,20]. It acts favorably both in terms of the recovery of the affected tissue and the short regeneration time for the three types of lesions, respectively nine days of treatment for incision and excision model and 12 days for the burn-type lesion. Moreover, the clinical results may be observed from the ninth day, and were confirmed later histologically, on the 21st, as shown in the histological images. The antimicrobial and bactericidal properties cited in the literature [5,6], were also demonstrated in the present study. These effects resulted from the anatomopathological analysis, which noted the presence of abscess and inflammatory infiltrate in the lesions of the untreated group and the one treated with only ointment base, and the lack of these characteristics for the batch treated with chitosan ointment.

It is known that too high a level of collagen can compromise the end result of the wound healing with formation of hypertrophic scars. Normal collagen production during the cicatrization process is essential for epithelial migration and post-injury proliferation [21]. In the present study, a normal collagenization was observed due to the use of chitosan that was included in the ointment base, which led to a restitutio ad integrum healing.

Chitosan molecules can induce a migration of inflammatory cells and fibroblasts, stimulating the production of cytokines and wound healing. Moreover, it is shown that wound healing with the aid of chitosan stimulates not only the migration and proliferation of fibroblasts, but also the production of collagen [6,21]. Fibroblasts stimulated by chitosan molecules secrete interleukin-8 (IL-8), which is an angiogenic and chemotactic factor of both cell types (endothelial and epidermal), which produce fibrosis and epithelialization [21,22].

By observing the collagenization process, from the third to ninth day of this experiment we noted that in the group treated with the chitosan ointment, the healing process progresses from inflammation of the epidermis and dermis to the stage of dermal collagenization in only six days for the incision-like lesion, and also for the excision-type lesion. For the burn-type lesion, in the same period of time, there is an evolution from inflammation in the hypodermis to the stage of important dermal collagenization due to the maturation of the granulation tissue, as confirmed by anatomopathological evidence. Therefore, in the case of the present study, chitosan acts both by stopping the inflammatory phenomenon and by stimulating the collagenization and maturation of the granulation tissue. Both effects were not found for the group treated with ointment base or for the untreated group.

It is also possible to deduce the effect that the chitosan-based ointment has on the normal regeneration processes, by observing that in the untreated group there were noticed phenomena of vacuolar degeneration and exocytosis even from the third day of the experiment. At the same time, also within the normal regeneration, we can say that the chitosan ointment intervenes beneficially on parakeratosis, which was observed in the case of the ointment base batch, on the third day, for the thermal injury type experiment.

In lyophilized egg whites, the active ingredients are proteins, zeaxanthin, lutein, riboflavin, selenium, choline, essential and non-essential amino acids [23]. Ovalbumin, protein contained in egg white (54%), forms a protective layer adherent to the lesion, inducing the regeneration of the affected skin tissue [8,24]. Not negligible is the fact that the lyophilized egg white alone included in the ointment base showed good effects in terms of wound healing. The preparation obtained with compounds biocompatible with the human body determined the regeneration of the affected skin tissue by forming a protective film adherent to the lesion.

In the present study, an ointment containing a combination of collagen, chitosan and lyophilized egg white was also used for wound healing. Fresh egg white is used in folk medicine in the treatment of lesions, especially in the heat burn injury, by direct application, forming a protective protein film on the surface of the lesion. The mixing of collagen with chitosan led to an increase in the release time at the level of the lesion, which allows a longer lasting action of the active ingredients. Based on literature data, the polymeric preparation obtained and tested in this experiment contains compounds biocompatible with the human body, with a role in regenerating the affected tissue, with hemostatic activity, and antimicrobial effect [5,25,26]. Moreover, the results obtained by testing ointments that involved the incorporation of the mixture of polymers in the ointment base, both clinically and by analyzing histological samples, reveals a superior therapeutic effect in terms of skin tissue regeneration. It was observed (for POL group) on the first day when the experimental models were made, for incision and excision lesions, an increased capacity of hemostasis. Moreover, the ointment intervenes favorably in the regeneration processes, noting the granulation tissue and, finally, a newly formed, young, maturing epithelium that was elastic and without traces of vicious healing, which is remarkable and noteworthy. The study opens a broad perspective for the formulation of new polymeric support types, which include bioactive compounds with controlled release system.

## 4. Materials and Methods

### 4.1. Ointments Preparation

The ointment base was prepared by mixing lanolin and vaseline in equal amounts on a water bath at 40 °C until a homogeneous base was obtained. The 0.5 g collagen polymer was solubilized in 10 mL of bidistilled water at 40 °C followed by the addition of 25 mL anhydrous glycerol. The solution thus obtained was incorporated into 100 g ointment base and thoroughly mixed (the ointment with collagen).

An amount of 0.24 g chitosan was dissolved in 30 mL apple honey vinegar and added to 100 g ointment base (the ointment with chitosan).

In order to obtain the ointment with lyophilized chicken egg white (*Gallus gallus domesticus*), the albumen of fresh eggs was separated from the yolk. The samples containing the egg white were frozen in Petri dishes and lyophilized. After the lyophilization process, the egg white was finely ground and 3 g were added to 100 g ointment base and mixed continuously (the ointment with lyophilized egg white). For the ointment based on total polymers, equal amounts of the three ointments described above were mixed.

### 4.2. Chemical Reagents

The ethyl alcohol, chitosan and collagen were purchased from Sigma-Aldrich (Steinheim, Germany), lanolin and vaseline were from Farma Chim (Ploiesti, Romania). The fresh eggs were obtained from a commercial source.

### 4.3. Rheological Characterization of Ointments

Rheological tests were carried out on a Physica MCR 501 (Anton Paar, Graz, Austria) modular rheometer equipped with a Peltier temperature control device. Parallel plate geometry (50 mm diameter) with serrated plates was used to avoid slippage of the sample. A thin layer of low viscosity silicone oil was applied on the edges of the sample to avoid moisture loss during studies. The rheological parameters of the samples were measured using oscillatory tests (amplitude sweep, frequency sweep, temperature sweeps) and rotational tests (flow curves). All experiments were performed at two different temperatures (25 °C and 37 °C). Rheological tests reproducibility was verified on three samples of each analyzed ointment.

### 4.4. Experimental Skin Lesions

The skin lesions were performed on laboratory adult male Wistar rats, with a body weight of 220–250 g fed a standard chow pellet diet and water ad libitum. The study included six Wistar rats groups with 7 animals per group as follows: NC group (negative control group–not treated), OB group–ointment base group (treated with the ointment base), CO group (treated with the collagen ointment), CH group (treated with the chitosan ointment), AL group (treated with ointment based on the lyophilized egg white, POL group (treated with the ointment based on the mixture of polymers).

The animals were anesthetized with Ketamine intraperitoneally (100 mg/kg) before inducing the wounds. The hairs on the dorsal part of the rats were shaved and cleaned with 70% alcohol. The treatment was applied topically once a day for 21 days. Each group of rats was housed in a separate cage with free access to standard laboratory diet and water.

Three experimental wound models (linear incision, circular excision, thermal burn) were applied on each animal. In this study the design of the lesions was based on previously described models [11,27,28].

The evaluated parameters were clinical examination, determination of lesion area, wound contraction rate, period of re-epithelialization and histological examination performed at days 0, 3, 9 and 21 of treatment. Therefore, a tissue sample was removed from the rats.

### 4.5. Wound Healing Evaluation Parameters

Circular lesion area (for excision wound model) was calculated according to the formula A = πr^2^. The elliptical area resulting from the lesion contraction process was calculated according to the formula:

π*a* × *b*/4 (1)

where *a* represented the high axis and *b* represented the small axis.

The wound contraction rate (WCR) was determined by the formula

(A_0_ − A_t_)/A_0_×100 (2)

where A_0_ is the original wound size (50.27 mm^2^) and A_t_ is the wound size on day 6 and 9, respectively.

Wound closure was considered the endpoint of the epithelialization process and days needed to complete epithelialization were considered the period of epithelialization.

### 4.6. Histological Examination

In order to observe the epithelialization process, tissue skin samples were removed from the rats with a 3 mm biopsy punch, after the rats were anesthetized intraperitoneally with ketamine (100 mg/kg). The samples were inserted in 10% buffered formalin for at least 24 h, progressively dehydrated in solutions containing an increasing percentage of ethanol (60, 80, 90, and 98% *v*/*v*), clarified with amylic alcohol and finally, embedded in paraffin under vacuum condition. Further, the skin tissue was sectioned at 5 µm thickness, deparaffinized, and stained with hematoxylin-eosin (HE).

The scoring of the dermal inflammatory infiltrate was performed based on scores from the scientific literature, adapted [29,30]. In order to establish the score, five microscopic fields with a magnification of 400× were examined, at the level of the papillary and reticular dermis and also at the hypodermis and striated muscle tissue and chosen as representative for each case. The value of the final score was represented by the average value of the five fields observed. A score of 0 (S0) represented no inflammatory infiltrate, score 1 (S1) mild/rare/occasional inflammatory infiltrate (<10 lymphocytes/HPF), score 2 (S2) moderate/focal inflammatory infiltrate (11–30 lymphocytes/HPF), score 3 (S3) severe inflammatory infiltrate (numerous lymphocytes > 30/HPF).

The evaluation of the thermal burn depths (D) was also achieved based on adapted scores from the literature as follows: D0–normal skin, D1–epithelial necrosis within the epidermis, the basement membrane remains intact, D2–necrosis of skin appendages and dermal connective tissue; D3–extensive necrosis within the hypodermic tissue [12,13].

### 4.7. Statistical Analysis

The data obtained from excision wound model were analyzed by one-way ANOVA followed by Bonferroni test. Statistical analysis was performed using SPSS version 15.0, (SPSS Inc., Chicago, IL, USA) where *p* < 0.05 was considered statistically significant.

## 5. Conclusions

The components of the ointments and their proportion directly influence the rheological characteristics of the pharmaceutical formulation. The samples exhibited time-independent dynamic moduli values, indicating the structural long-term stability of the ointments. These results suggest that the viscoelastic behavior of ointments can be adjusted by choosing the right ratio of components, thus allowing the preparation of ointments for specific applications.

All four ointments tested in this experiment have been proven effective in the incision, excision, and thermal burn lesions. The incorporation of chitosan, collagen, and lyophilized egg white in the same formulation (total polymers-based ointment), through synergistic effects, led to the formation of a protective film, hemostasis on affected tissues, and cell proliferation by the property of generating new tissue. Also, normal skin tissue regeneration and normal collagenization were observed. The ointments stopped the effects of vacuolar degeneration, exocytosis, and parakeratosis. They also exhibited antiinflammatory effect and favorable results on collagenization and maturation of tissue, as proven by the morpho-pathological analyses.

## Figures and Tables

**Table 1:** The values of zero shear viscosity (Carreau-Yasuda model).

Sample	Zero Shear Viscosity (Pa·s)	
	25 °C	37 °C
AL	21,908	702.92
CH	97,898	1435.8
CO	19,147	1190.3
POL	27,964	1374.2

AL—ointment with ovalbumin from lyophilized egg white; CH—chitosan ointment; CO—collagen ointment; POL—ointment containing mixture of AL, CH and CO.

**Table 2:** Evaluation of wound area, re-epithelialization area and wound. Contraction rate (WCR) for the excision model.

Experimental Groups	Wound Area (mm^2^)			Re-Epithelialization Area (mm^2^)	WCR (%) (Mean ± SEM)	
	Day 0	Day 6	Day 9	Day 12	Day 6	Day 9
NC Group	64 (8 × 8)	61.6 (7.7 × 8)	56.25 (7.5 × 7.5)	-	5.78 ± 1.86	14.85 ± 2.95
OB Group	64 (8 × 8)	45.5 (6.5 × 7)	40.8 (6 × 6.8)	-	26.74 ± 2.13 *	33.79 ± 2.21 *
CO Group	64 (8 × 8)	39 (6.5 × 6)	6 (2 × 3)	2 × 2	40.00 ± 0.94	89.53 ± 0.68
CH Group	64 (8 × 8)	36 (6 × 6)	4 (2 × 2)	2 × 1	44.69 ± 0.83	93.43 ± 0.33
AL Group	64 (8 × 8)	22.5 (4.5 × 5)	2 (1 × 2)	1 × 1	64.06 ± 0.45	97.14 ± 0.14
POL Group	64 (8 × 8)	25 (5 × 5)	6 (2 × 3)	1 × 1	60.42 ± 0.52	91.46 ± 0.45

* *p* < 0.001 NC group (negative control group—not treated), OB group—ointment base group (treated with the ointment base), CO group (treated with the collagen ointment), CH group (treated with the chitosan ointment), AL group (treated with the ointment based on the lyophilized egg white, POL group (treated with the ointment based on the mixture of polymers).

## Data Availability

The data presented in this study are available on request from the corresponding author.

## Supplementary Materials

The following are available online at https://www.mdpi.com/article/10.3390/ph14050465/s1, Figure S1: Macroscopic pictures of the cutaneous lesions.

## References

[1] Murray R.Z., West Z.E., Cowin A.J., Farrugia B.L. (2019). Development and use of biomaterials as wound healing therapies. Burn. Trauma.

[2] Naomi R., Ratanavaraporn J., Fauzi M.B. (2020). Comprehensive Review of Hybrid Collagen and Silk Fibroin for Cutaneous Wound Healing. Materials.

[3] Santhanam R., Rameli M.A.P., Al Jeffri A., Ismail W.I.W. (2020). Bovine Based Collagen Dressings in Wound Care Management. J. Pharm. Res. Int..

[4] Negut I., Dorcioman G., Grumezescu V. (2020). Scaffolds for Wound Healing Applications. Polymers.

[5] Moeini A., Pedram P., Makvandi P., Malinconico M., D’Ayala G.G. (2020). Wound healing and antimicrobial effect of active secondary metabolites in chitosan-based wound dressings: A review. Carbohydr. Polym..

[6] El-Hack M.E.A., El-Saadony M.T., Shafi M.E., Zabermawi N.M., Arif M., Batiha G.E., Khafaga A.F., El-Hakim Y.M.A., Al-Sagheer A.A. (2020). Antimicrobial and antioxidant properties of chitosan and its derivatives and their applications: A review. Int. J. Biol. Macromol..

[7] Lončarević A., Ivanković M., Rogina A. (2017). Lysozyme-Induced Degradation of Chitosan: The Characterisation of Degraded Chitosan Scaffolds. J. Tissue Repair Regen..

[8] Li Y., Zhang H., Zhang S., Yan X., Shao Y., Jiang Y. (2020). Egg White peptide KPHAEVVLR promotes skin fibroblasts migration and mice skin wound healing by stimulating cell membrane Hsp90α secretion. Process. Biochem..

[9] Ibanescu C., Danu M., Nanu A., Lungu M., Simionescu B. (2010). Stability of disperse systems estimated using rheological, oscillatory shear tests. Rev. Roum. Chim..

[10] Merlusca I.P., Ibanescu C., Tuchilus C., Danu M., Atanase L.I., Popa I.M. (2019). Characterization of neomycin-loaded xanthan-chitosan hydrogels for topical applications. Cellul. Chem. Technol..

[11] Andritoiu C.V., Andriescu C.E., Ibanescu C., Lungu C., Ivanescu B., Vlase L., Havarneanu C., Popa M. (2020). Effects and Characterization of Some Topical Ointments Based on Vegetal Extracts on Incision, Excision, and Thermal Wound Models. Molecules.

[12] Medina J.L., Fourcaudot A.B., Sebastian E.A., Shankar R., Brown A.W., Leung K.P. (2018). Standardization of deep partial-thickness scald burns in C57BL/6 mice. Int. J. Burn. Trauma.

[13] Wohlsein P., Peters M., Schulze C., Baumgärtner W. (2016). Thermal Injuries in Veterinary Forensic Pathology. Vet. Pathol..

[14] El-Sayed Y.S. (2016). Time Course of Histomorphologic Features during Chronic Burn Wound Healing. Forensic Med. Anat. Res..

[15] Lim Y.-S., Ok Y.-J., Hwang S.-Y., Kwak J.-Y., Yoon S. (2019). Marine Collagen as A Promising Biomaterial for Biomedical Applications. Mar. Drugs.

[16] Odermatt E.K., Steuer H., Lembert N. (2017). Efficacy of a collagen hemostat versus a carrier-bound fibrin sealant. J. Thrombo. Cir..

[17] Chattopadhyay S., Raines R.T. (2014). Collagen-based biomaterials for wound healing. Biopolymers.

[18] Zeltz C., Gullberg D. (2016). The integrin–collagen connection—A glue for tissue repair?. J. Cell Sci..

[19] Pan H., Fan D., Cao W., Zhu C., Duan Z., Fu R., Li X., Ma X. (2017). Preparation and Characterization of Breathable Hemostatic Hydrogel Dressings and Determination of Their Effects on Full-Thickness Defects. Polymers.

[20] Tripathi D., Rastogi K., Tyagi P., Rawat H., Mittal G., Jamini A., Singh H., Tyagi A. (2021). Comparative Analysis of Collagen and Chitosan-based Dressing for Haemostatic and Wound Healing Application. AAPS PharmSciTech.

[21] Patrulea V., Ostafe V., Borchard G., Jordan O. (2015). Chitosan as a starting material for wound healing applications. Eur. J. Pharm. Biopharm..

[22] Ishihara M., Nakanishi K., Ono K., Sato M., Kikuchi M., Saito Y., Yura H., Matsui T., Hattori H., Uenoyama M. (2002). Photocrosslinkable chitosan as a dressing for wound occlusion and accelerator in healing process. Biomaterials.

[23] Fernandez M.L., Lemos B., Wu J. (2019). Eggs are natural functional food. Eggs as Functional Foods and Nutraceuticals for Human Health.

[24] Jalili-Firoozinezhad S., Filippi M., Mohabatpour F., Letourneur D., Scherberich A. (2020). Chicken egg white: Hatching of a new old biomaterial. Mater. Today.

[25] Gaspar-Pintiliescu A., Stanciuc A.-M., Craciunescu O. (2019). Natural composite dressings based on collagen, gelatin and plant bioactive compounds for wound healing: A review. Int. J. Biol. Macromol..

[26] Augustine R., Rehman S.R.U., Ahmed R., Zahid A.A., Sharifi M., Falahati M., Hasan A. (2020). Electrospun chitosan membranes containing bioactive and therapeutic agents for enhanced wound healing. Int. J. Biol. Macromol..

[27] Süntar I.P., Akkol E.K., Yılmazer D., Baykal T., Kırmızıbekmez H., Alper M., Yeşilada E. (2010). Investigations on the in vivo wound healing potential of Hypericum perforatum L. J. Ethnopharmacol..

[28] De Mesquita C.J.G., Leite J.A., Fechine F.V., de Rocha J.L.C., Leite J.G., Leite Filho J.A., Barbosa Filho R.A. (2010). Effect of imiquimod on partial-thickness burns. Burns.

[29] Clark M.D., Peters-Kennedy J., Scott D.W. (2013). Resident lymphocytes in the epidermis and adnexal epithelia of normal dorsolateral thorax of alpacas. Can. J. Vet. Res..

[30] Agbaje M., Rutland C.S., Maboni G., Blanchard A., Bexon M., Stewart C., Jones M.A., Totemeyer S. (2018). Novel inflammatory cell infiltration scoring system to investigate healthy and footrot affected ovine interdigital skin. PeerJ.

