# Supplementary material for: Evaluation of the Wound Healing Potential of Some Natural Polymers on Three Experimental Models
Source: Pharmaceuticals (Basel). 2021 May 14;14(5):465. doi: 10.3390/ph14050465 (PMC8156046; doi:10.3390/ph14050465)
Supplement: Supplementary file 1 [file pharmaceuticals-14-00465-s001.zip › pharmaceuticals-1183506-supplementary.pdf]

Figure S1. Macroscopic pictures of the cutaneous lesions.

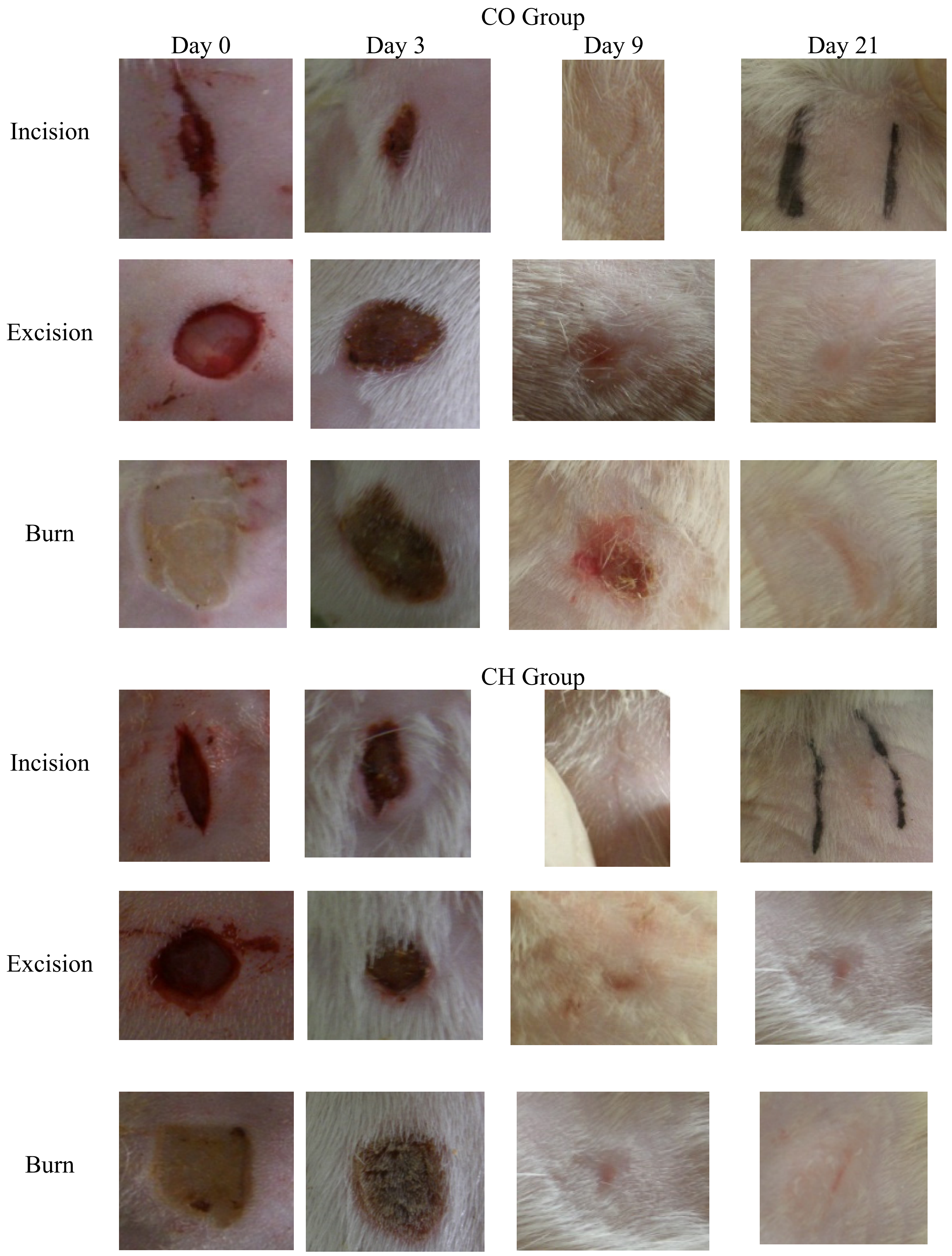

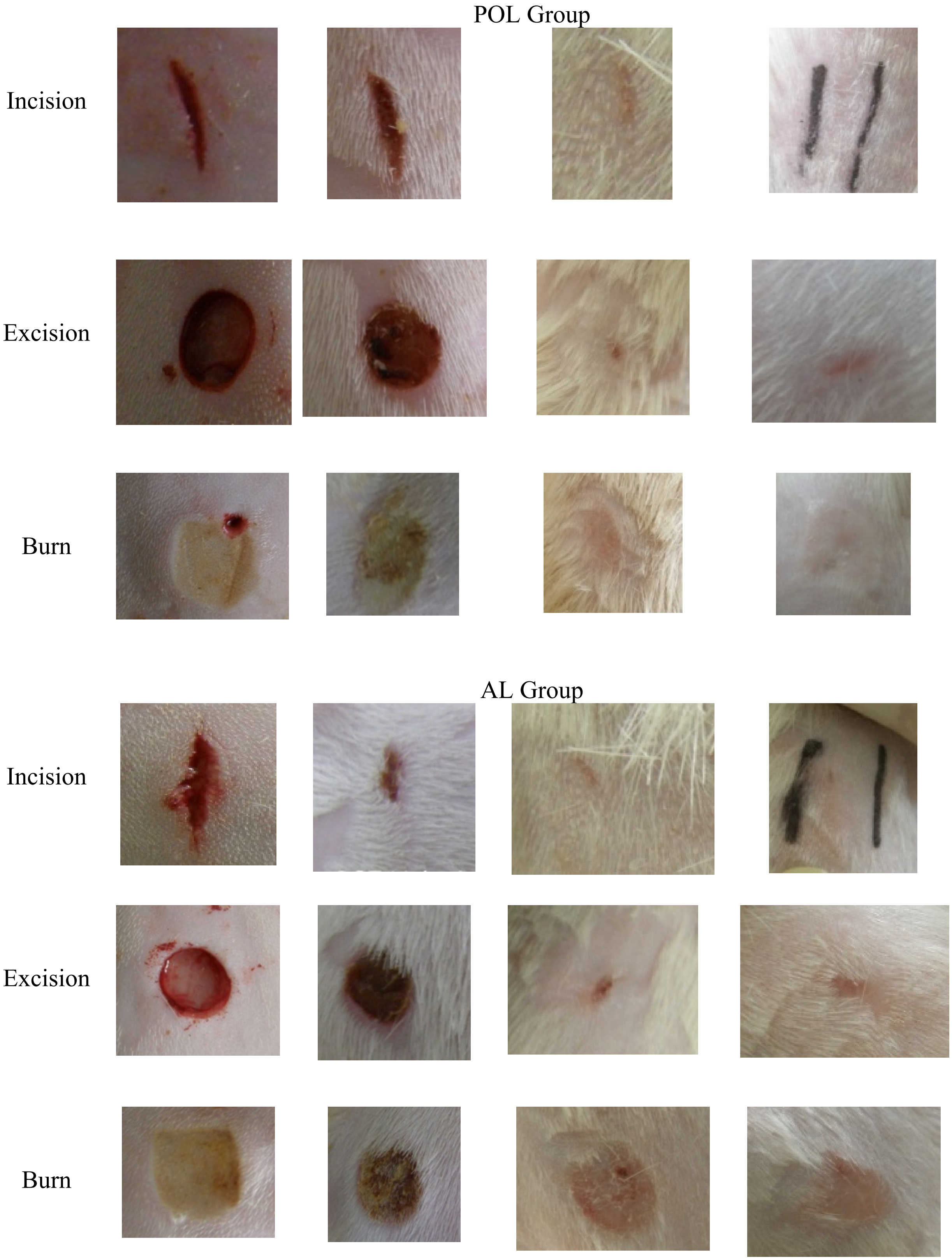
POL Group
Incision
Excision
Burn
AL Group
Incision
Excision
Burn

CO group (treated with the collagen ointment), CH group (treated with the chitosan ointment), POL group (treated with the ointment based on the mixture of polymers), AL group (treated with the ointment based on the lyophilized egg white)
